# Effects of the COVID-19 Pandemic on Treatment Efficiency for Traumatic Brain Injury in the Emergency Department: A Multicenter Study in Taiwan

**DOI:** 10.3390/jcm10225314

**Published:** 2021-11-15

**Authors:** Carlos Lam, Ju-Chuan Yen, Chia-Chieh Wu, Heng-Yu Lin, Min-Huei Hsu

**Affiliations:** 1 Emergency Department, Wan Fang Hospital, Taipei Medical University, Taipei 11696, Taiwan; lsk@w.tmu.edu.tw (C.L.); setfreej@gmail.com (C.-C.W.); 2 Department of Emergency, School of Medicine, College of Medicine, Taipei Medical University, Taipei 11030, Taiwan; 3 Department of Ophthalmology, Taipei City Hospital, Renai Branch, Taipei 10629, Taiwan; m701061@tmu.edu.tw; 4 Graduate Institute of Biomedical Informatics, College of Medical Technology, Taipei Medical University, Taipei 11030, Taiwan; 5 School of Medicine, College of Medicine, Taipei Medical University, Taipei 11030, Taiwan; b101105091@tmu.edu.tw; 6 Graduate Institute of Data Science, College of Management, Taipei Medical University, Taipei 11030, Taiwan

**Keywords:** COVID-19 pandemic, treatment efficiency, traumatic brain injury, emergency department

## Abstract

The coronavirus disease 2019 (COVID-19) pandemic has impacted emergency department (ED) practice, including the treatment of traumatic brain injury (TBI), which is commonly encountered in the ED. Our study aimed to evaluate TBI treatment efficiency in the ED during the COVID-19 pandemic. A retrospective observational study was conducted using the electronic medical records from three hospitals in metropolitan Taipei, Taiwan. The time from ED arrival to brain computed tomography (CT) and the time from ED arrival to surgical management were used as measures of treatment efficiency. TBI treatment efficiencies in the ED coinciding with a small-scale local COVID-19 outbreak in 2020 (P1) and large-scale community spread in 2021 (P2) were compared against the pre-pandemic efficiency recorded in 2019. The interval between ED arrival and brain CT was significantly shortened during P1 and P2 compared with the pre-pandemic interval, and no significant delay between ED arrival and surgical management was found, indicating increased treatment efficiency for TBI in the ED during the COVID-19 pandemic. Minimizing viral spread in the community and the hospital is vital to maintaining ED treatment efficiency and capacity. The ED should retain sufficient capacity to treat older patients with serious TBI during the COVID-19 pandemic.

## 1. Introduction

Due to geographic proximity with China, hospitals in Taiwan rapidly prepared for the impending arrival of the coronavirus disease 2019 (COVID-19) infection soon after the outbreak was first reported in Wuhan, China, in 2019 [1]. Although the number of COVID-19 cases reported in European countries began to grow exponentially [2,3], the Taiwan Centers for Disease Control (CDC) implemented strict border control and infection control measures to prevent virus transmission [4]. Controlled access to medical facilities was enforced, and a screening station was established outside of the emergency department (ED) to secure hospitals [5]. The rapid response by the CDC and the cooperation by the population resulted in outstanding performance for controlling the COVID-19 pandemic in Taiwan in 2020 [6].

In contrast to many countries that suffered from healthcare system damage due to severe community and hospital spread of the virus, the hospitals in Taiwan were able to continuously provide regular services after the pandemic was declared in 2020. People’s daily lives remained relatively unchanged until the barricade was broken through in 2021. A cluster of COVID-19 infections was identified in metropolitan Taipei in May 2021, and the infection rapidly spread across many communities on the island [7]. A ban against large gatherings and the semi-lockdown of cities were immediately implemented when the number of confirmed cases escalated from 1199 to 4917 over a two-week period, resulting in a substantial decrease in outdoor activities.

After the outbreak of community infection, the continuous emergence of COVID-19 pneumonia forced hospitals in Taiwan to restrict their daily workloads to ensure the sufficient availability of human resources in dedicated COVID-19 wards [8,9]. These highly contagious patients also profoundly disturbed the daily workflow in the ED [10].

A significant decrease in ED visits for injury was observed in many countries after the pandemic was declared [11,12,13,14,15]. Although no widespread transmission of COVID-19 infection was reported in Taiwan in 2020, a similar trend in decreased ED visits was reported in Taiwan [16,17]. The drop in ED visits for injury was even more profound following the detection of community spread in 2021. Traumatic brain injury (TBI) is one of the most common diseases treated in the ED, and nearly 80% of treated cases are classified as mild injuries. Although the number of TBI cases has steadily increased over time [18,19], the number of TBI cases in the ED declined significantly during the COVID-19 pandemic, which is known as the “coronavirus lockdown effect” [20]. A study in the United Kingdom showed that referrals for TBI decreased by 49.6% [21], and the decreases reported in India, the Netherlands, and Ireland were 60%, 36%, and 17.1%, respectively [20,22,23]. For TBI patients, a brain computed tomography (CT) scan is indispensable to detect the presence of brain hemorrhage. Previous studies showed that the average daily number of brain CT scans decreased during the pandemic. However, the proportion of cases with acute findings rose significantly [24]. A similar trend was reported for other injuries and diseases treated in the ED [14,25].

The restriction of the hospital’s human resources in the operation room also impacted the treatment of the TBI during the pandemic. In addition, the processes implemented to determine COVID-19 infection status also delayed operations, which may have contributed to the increased mortality rate observed during the lockdown period [26,27]. The current consensus recommendation is that all medical personnel should wear appropriate protective equipment when performing surgery on patients with suspected COVID-19 infection [28,29]. These infection control precautions likely complicated the surgery procedures.

The emergence of the Delta variant indicated that the battle against the COVID-19 pandemic would be continuous. In Taiwan, only one wave of community spread was reported one year after the pandemic declaration, representing a course that differed from most other countries. Therefore, our study aimed to evaluate the impacts of the COVID-19 pandemic on the treatment efficiency of TBI in the ED. The pre-pandemic era was compared with a period of small-scale local infection during the early stages of the COVID-19 pandemic in 2020 and with the period marked by large-scale community spread that occurred after May 2021. The results of this study provide important information for the staff of EDs and neurosurgery departments and for hospital administration regarding the maintenance of efficiency and the appropriate management of TBI in the ED during the COVID-19 pandemic.

## 2. Materials and Methods

### 2.1. Data Source

A retrospective observational study was conducted using the Clinical Research Database (CRD) of the Taipei Medical University. The CRD contains the electronic medical records from the following three affiliated teaching hospitals: Taipei Medical University Hospital, Wan Fang Hospital, and Shuang Ho Hospital. These three hospitals are located in metropolitan Taipei and are accredited as advanced emergency responsibility hospitals that provide comprehensive care for major trauma patients.

We extracted data for ED visits, brain CT scans, and brain operations from the CRD between 1 January and 31 July 2019, 2020, and 2021. Identifiable information from these hospital data was encrypted to ensure patient confidentiality. The Institutional Review Board of Taipei Medical University approved this study (No.: N202106027).

### 2.2. Sample Selection

**Figure 1:**
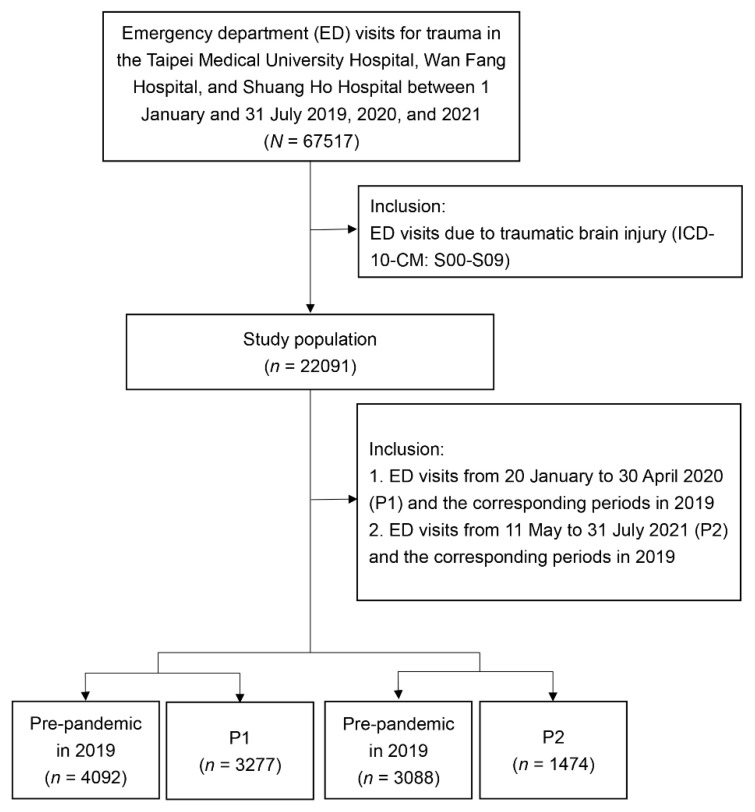
Sample selection procedure from the CRD of Taipei Medical University. CRD, Clinical Research Database; ED, emergency department; P1, 20 January to 30 April 2019; P2, 11 May to 31 July 2021. Pre-pandemic period refers to the same span from 2019.

We selected all ED visits due to traumatic injury between 1 January and 31 July 2019, 2020, and 2021 and only included those associated with the International Classification of Diseases, Tenth Revision, Clinical Modification (ICD-10-CM) codes for trauma: S00–S99. TBI was identified by the ICD-10 codes S00–S09. Figure 1 presents the flow chart for sample selection.

All trauma-related ED visits at participating hospitals during the period associated with small-scale local infection from January 20 to 30 April 2020 (period one, P1) and the period associated with large-scale community spread from 11 May to 31 July 2021 (period two, P2) were included in our study. The treatment efficiencies for TBI in the ED during P1 and P2 were compared with corresponding periods in 2019 (pre-pandemic).

### 2.3. Measurement

Collected characteristics of the sample included sex, age, triage level, and TBI patterns. The triage level was categorized as critical (levels I and II), urgent (level III), and less urgent (levels IV and V). The TBI patterns included mild head injury (ICD-10-CM: S00, S01, S09, and S06.0) and serious head injury (ICD-10-CM: S06.1–S06.9).

The time from ED arrival to the completion of brain CT and the time from ED arrival to the start of brain operation were used as proxies to represent treatment efficiency for TBI. We only included brain operations coded as urgent in the ED and performed within 24 h after ED arrival.

### 2.4. Statistical Analysis

We first plotted weekly ED visits from 1 January to 31 July 2019, 2020, and 2021 to demonstrate the numbers of yearly ED visits due to trauma, TBI, mild head injury, and serious head injury. We also plotted the numbers and rates of brain CT scans and brain operations among TBI-related ED visits.

The sample characteristics, TBI patterns, numbers of brain CT scans, and numbers of operations during P1 and P2 were separately compared with their corresponding pre-pandemic values using the Chi-square and Wilcoxon rank-sum tests. Kaplan–Meier survival analysis was used to evaluate time-to-event data (time to brain CT), and differences were evaluated using a nonparametric log-rank test. A 2-sided *p*-value of <0.05 was considered statistically significant. All statistical analyses were performed using SAS version 9.4 (SAS Institute, Cary, NC, USA).

## 3. Results

**Figure 2:**
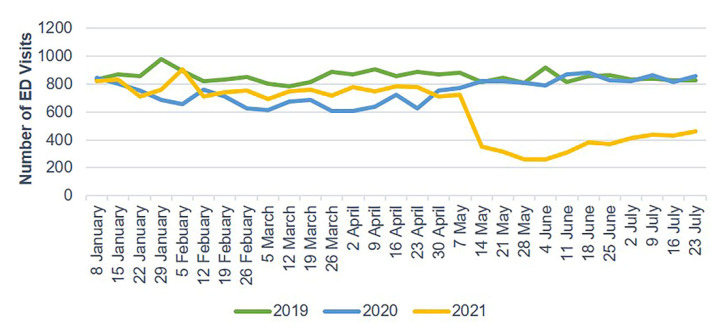
Weekly trauma-related ED visits from January to July 2019, 2020, and 2021. ED, emergency department.

**Figure 3:**
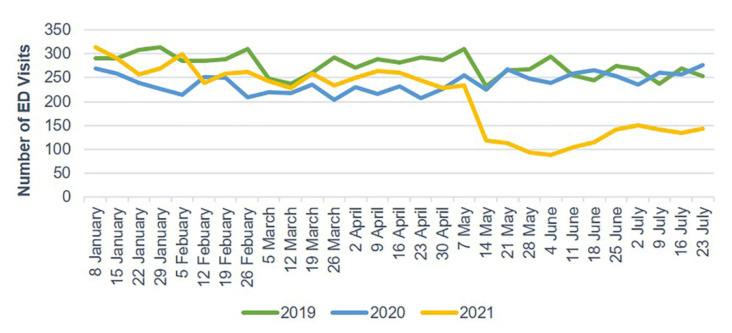
Weekly TBI-related ED visits from January to July 2019, 2020, and 2021. ED, emergency department; TBI, traumatic brain injury.

**Figure 4:**
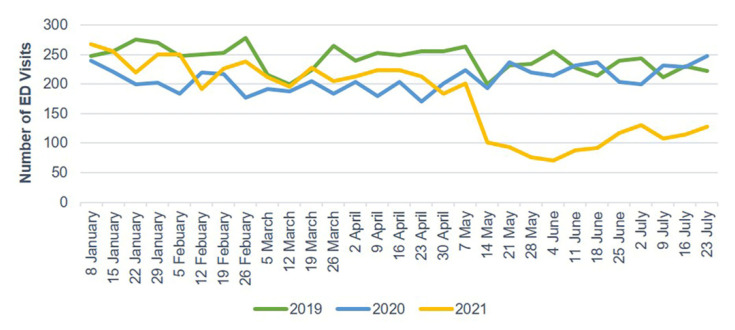
Weekly ED visits due to mild head injury from January to July 2019, 2020, and 2021. ED, emergency department.

**Figure 5:**
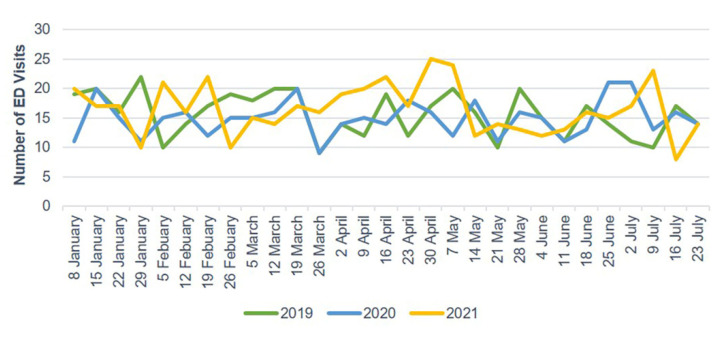
Weekly ED visits due to serious head injury from January to July 2019, 2020, and 2021. ED, emergency department.

**Figure 6:**
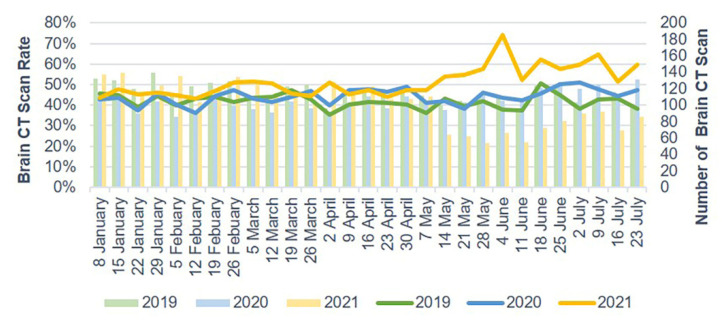
Brain CT scan rate for TBI in the ED from January to July 2019, 2020, and 2021. CT scan, computerized tomography scan; ED, emergency department; TBI, traumatic brain injury.

**Figure 7:**
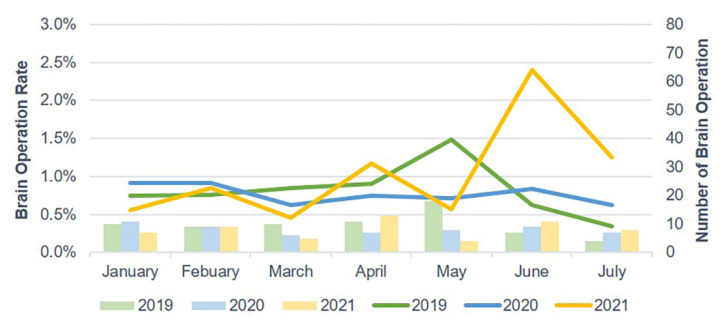
Brain operation rate for TBI in the ED from January to July 2019, 2020, and 2021. ED, emergency department; TBI, traumatic brain injury.

The number of ED visits due to trauma and TBI each week decreased starting in late January 2020 and gradually increased after 30 April 2020. In 2021, the weekly number of ED visits due to trauma and TBI sharply dropped starting on 14 May (Figure 2 and Figure 3). Mild head injuries were reduced during P1 and P2 compared with the pre-pandemic period (Figure 4). However, the drop in serious head injuries was insignificant in P1 (Figure 5). Although the number of brain CT scans performed for TBI decreased in P2, the rate of brain CTs rose sharply (Figure 6). The rate of brain operations also significantly increased in P2 (Figure 7).

The numbers of ED visits were 3277 during P1 and 4092 during the corresponding pre-pandemic period and 1474 during P2 and 3088 during the corresponding pre-pandemic period. The distribution of intracranial injuries (S06.0–S06.9) and neurosurgical procedures and their frequency in the pre-pandemic and pandemic periods are shown in the Supplementary Figure S1 and Table S1.

Table 1 shows a comparison between the proportions of TBI-related ED visits and TBI injury patterns before and after the COVID-19 pandemic. The proportion of TBI-related ED visits in P2 was significantly higher than that in the corresponding pre-pandemic period in 2019 (33.57% vs. 31.27%, *p* = 0.007). No significant difference was noted in the proportions of TBI-related visits between P1 and the pre-pandemic period. The proportion of mild head injury was significantly reduced during P2 compared with the respective pre-pandemic period in 2019 (83.22% vs. 87.01%, *p* = 0.001), whereas the proportions of serious head injury significantly increased in P2 compared with the respective pre-pandemic period (11.30% versus 5.79%, *p* < 0.0001). No such change was found in P1.

Table 2 shows a comparison of the characteristics of TBI samples before and after the COVID-19 pandemic. The ages of patients who visited the ED for TBI during P1 and P2 were significantly older (P1: 44 years vs. 42 years, *p* < 0.001; P2: 54 years versus 42 years, *p* < 0.0001) than those during the respective pre-pandemic periods. A comparison of the triage levels also showed significant increases in critical TBI during P1 and P2 (P1: 13.61% vs. 11.93%, *p* < 0.001; P2: 22.59% vs. 11.82%, *p* < 0.0001).

Table 3 shows a comparison between the treatment efficiencies for TBI-related ED visits before and after the COVID-19 pandemic. During P1 and P2, the times from ED arrival to brain CT were significantly shorter than for the respective pre-pandemic periods (P1: 22 min versus 30 min, *p* < 0.0001; P2: 21 min vs. 27 min, *p* < 0.0001). No significant change was observed in the time from ED arrival to brain operation for either P1 or P2 compared with the respective pre-pandemic period.

**Figure 8:**
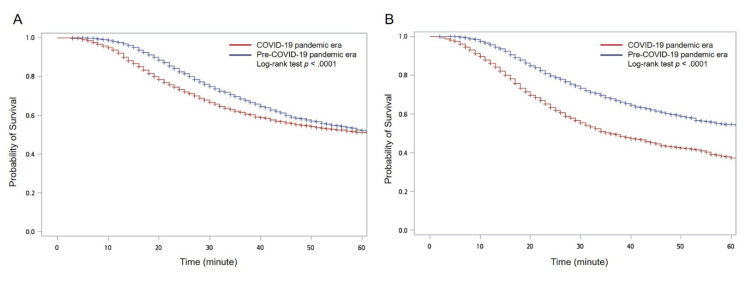
Kaplan–Meier curves with log-rank test for the time from emergency department (ED) arrival to brain computed tomography (CT) for traumatic brain injury (TBI)-related ED visits between the COVID-19 pandemic era and the pre-COVID-19 pandemic era during (**A**) P1 and (**B**) P2.

The Kaplan–Meier curves also showed significant differences in the time from ED arrival to brain CT between the COVID-19 pandemic era (stratified by P1 and P2) and the pre-COVID-19 pandemic era (Figure 8).

## 4. Discussion

At the beginning of the pandemic, in 2020, the number of TBI cases treated by the ED declined, consistent with reports from foreign countries [30,31]. This decrease has been attributed to a reduction in outdoor activities, which led to a decrease in road traffic injuries. A significant increase in the ages of TBI patients treated by the ED was observed because most of those injured due to household activities, such as accidental falls, are older adults. During this period, the reduction in outdoor activities was primarily the result of spontaneous changes in behavior in response to reports by the mass media. After a period during which no significant viral spread was reported, the population’s activities eventually returned to pre-pandemic levels. Consistently, the TBI numbers reported for the second quarter of 2020 gradually returned to the levels reported before the outbreak.

The outbreak in May 2021 (P2) was associated with a completely different pattern, with a large-scale community infection that spread across many communities [32]. The relevant authorities immediately banned large gatherings and implemented a city-wide semi-lockdown strategy to stop the spread [7]. During P2, outdoor activities and commuting were severely restricted, which was associated with a sharp decrease in TBI numbers, and the increase in the average patient age during this period was more pronounced than that observed for P1, indicating that the outdoor activities among younger adults were almost completely stopped, resulting in an increase in the proportion of TBI cases among older adults.

The COVID-19 outbreak impacted TBI patterns in the ED, associated with a decrease in mild and serious head injuries treated during P1 and P2 compared with 2019. The decrease in P2 was more obvious compared with P1. These results showed the effects of the city-wide semi-lockdown strategy during the large-scale community spread of the virus. During P2, the relevant authorities banned large gatherings, including school and work. Since most road traffic injuries in Taiwan are mild injuries [33], the semi-lockdown strategy during P2 restricted commuting, resulting in a sharp decline in the number of mild head injuries treated in the ED.

Because the reduction in overall trauma cases was small during P1, no significant changes in TBI proportions were noted compared with the proportions in 2019. However, the overall number of trauma cases treated in the ED declined significantly during P2, resulting in an increase in the proportion of TBI cases. The reduced commuting in P2 increased the proportions of serious head injuries. Although no such change was shown in P1, the proportions of patients who arrived at the ED in critical condition (triage levels I and II) increased during both P1 and P2, and the proportion of critical cases reported during P2 was almost double that for 2019. Therefore, the ED should maintain sufficient capacity to treat critical patients.

The outbreak also impacted brain CT execution in the ED. The number of brain CT scans performed during P1 and P2 decreased compared with the number performed in 2019. However, the decrease in P2 was more obvious compared with P1. The lockdown strategy sharply reduced the occurrence of mild head injury in P2, causing a significant rise in the proportions of brain CT scans.

When the COVID-19 outbreak was first reported in December 2019, all hospitals in Taiwan responded immediately. Access control was used to prevent high-risk patients from entering the hospital, and patient visiting activities were also banned [7]. Outside of the ED, screening stations were established to divert patients into low-, medium-, and high-risk areas for treatment. All ED staff, including emergency medical technicians, routinely used personal protective equipment, such as face shields, surgical gowns, and N95 masks [34]. As a result of these measures, no spread of COVID-19 infection has been reported in hospitals and EDs. Brain CT scans ordered for low-risk patients were performed as before, and only local disinfection was required after the examination. Therefore, the majority of TBI cases in the ED were examined without delay. Due to the preservation of ED capacity and the decline in TBI numbers, the time interval between ED arrival and brain CT performance was significantly shortened during P1.

Due to the lack of community or hospital COVID-19 spread during P1, patients who entered the operating room from a low-risk area in the ED were only submitted to a COVID-19 antigen test. The operating room staff used the same personal protective equipment required by ED staff, and most brain operations were performed similarly to the pre-pandemic period. No significant delay between ED arrival and brain operation was noted during P1.

During P2, clusters of infections in several communities were serious, and deaths increased daily, causing large psychological and behavioral impacts on society. People substantially reduced hospital visits due to fear of contacting infected patients. The continuous presentation of patients with COVID-19 pneumonia resulted in a huge burden on human resources for hospitals as the medical staff was increasingly diverted to treat COVID-19 patients [35]. The proper protection of the ED workforce allowed for the maintenance of treatment capacity. The COVID-19 PCR test was extensively used to detecting asymptomatic infections. For patients who required surgery, a rapid respiratory panel was universally used to reduce waiting times. ED staff used N100 masks or powered air-purifying respirators due to the extremely high probability of viral transmission when treating infected patients. For those COVID-19 patients who required CT scans or surgery, all staff members in contact with the patients, including ED physicians and neurosurgeons, were required to use full protection, including an isolation suit. The field exposed to the patient was treated according to a thorough disinfection procedure using bleach and alcohol.

These infection prevention measures delayed treatment in the ED. However, due to the sharp decline in the number of TBI cases and the preservation of the ED’s treatment capacity, the execution time for brain CT scans was significantly shortened, and the waiting time for brain operations did not increase. These results showed that the treatment efficiency for TBI in the ED increased during P2. During an outbreak of community spread, the proportions of TBI cases requiring brain CT and brain operations increased. Therefore, medical centers should maintain sufficient treatment capacity in the ED and neurosurgery departments to allow for the treatment of serious head injuries during COVID-19 outbreaks with community spread [20].

The multicenter approach strengthened the generalizability of our findings. However, community spread during P2 was concentrated in certain communities rather than evenly distributed. Therefore, the impacts of the pandemic on treatment efficiency were influenced by the locations of the hospitals. In addition, the study period only included the three months of the outbreak. During this period, the decrease in the total number of serious head injuries may bias the statistical results. Finally, the COVID-19 pandemic in Taiwan was well controlled. The number of patients infected by the virus was limited, and the health care system was not burdened to the same extent as in many other countries. Therefore, extrapolation of the results to other settings may be difficult.

## 5. Conclusions

The COVID-19 pandemic had a significant impact on the treatment efficiency for TBI in the ED. The impacts of preventing large gatherings and the city-wide semi-lockdown after a COVID-19 outbreak with community spread differed from impacts of self-initiated reductions in outdoor activities due to social panic during the early stages of the pandemic. Minimizing the spread of COVID-19 in the community and in hospitals and protecting ED capacity is vital to maintaining treatment efficiency for TBI. The proportion of older patients and the proportion of serious head injuries increase when overall numbers of TBI decline due to decreased participation in outdoor activities and commuting. Therefore, the ED and neurosurgery departments should retain sufficient capacity to treat these patients during a pandemic outbreak.

## Figures and Tables

**Table 1:** Comparison of the proportions of TBI-related ED visits and injury patterns before and after the COVID-19 pandemic.

Variable	Pre-Pandemic Period Corresponding to P1		P1		*p*	Pre-Pandemic Period Corresponding to P2		P2		*p*
	*n*	%	*n*	%		*n*	%	*n*	%	
Trauma population										
TBI					0.472					0.007
No	8318	67.00	6523	66.55		6800	68.73	2925	66.43	
Yes	4096	33.00	3279	33.45		3094	31.27	1478	33.57	
TBI population										
Mild head injury					0.083					0.001
No	505	12.33	449	13.69		402	12.99	248	16.78	
Yes	3591	87.67	2830	86.31		2692	87.01	1230	83.22	
Serious head injury					0.089					<0.0001
No	3864	94.34	3062	93.38		2915	94.21	1311	88.70	
Yes	232	5.66	217	6.62		179	5.79	167	11.30	

TBI, traumatic brain injury; ED, emergency department; COVID-19, coronavirus disease 2019; P1, January to 30 April 2019; P2, 11 May to 31 July 2021. Pre-pandemic period refers to the same span from 2019.

**Table 2:** Comparison of the TBI sample characteristics before and after the COVID-19 pandemic.

Variable	Pre-Pandemic Period Corresponding to P1 (*n* = 4092)		P1 (*n* = 3277)		*p*	Pre-Pandemic Period Corresponding to P2 (*n* = 3088)		P2 (*n* = 1474)		*p*
	*n*	%	*n*	%		*n*	%	*n*	%	
Sex					0.403					0.117
Female	1808	44.18	1416	43.21		1417	45.89	640	43.42	
Male	2284	55.82	1861	56.79		1671	54.11	834	56.58	
Age (years), median (IQR)	42	(19–66)	44	(22–68)	<0.001	42	(19–66)	54	(29–73)	<0.0001
Age (years)					0.001					<0.0001
0–14	812	19.84	537	16.39		620	20.08	188	12.75	
15–24	522	12.76	397	12.11		409	13.24	128	8.68	
25–44	833	20.36	728	22.22		598	19.37	274	18.59	
45–64	857	20.94	683	20.84		632	20.47	347	23.54	
65+	1068	26.10	932	28.44		829	26.85	537	36.43	
Triage					<0.001					<0.0001
Critical (Levels I and II)	488	11.93	446	13.61		365	11.82	333	22.59	
Urgent (Level III)	3479	85.02	2772	84.59		2619	84.81	1120	75.98	
Less urgent (Levels IV and V)	125	3.05	59	1.80		104	3.37	21	1.42	

IQR, interquartile range. TBI, traumatic brain injury; COVID-19, coronavirus disease 2019; P1, January to 30 April 2019; P2, 11 May to 31 July 2021. Pre-pandemic period refers to the same span from 2019.

**Table 3:** Comparison of treatment efficiencies for TBI-related ED visits before and after the COVID-19 pandemic.

Variable	Pre-Pandemic Period Corresponding to P1 (*n* = 4092)		P1 (*n* = 3277)		*p*	Pre-Pandemic Period Corresponding to P2 (*n* = 3088)		P2 (*n* = 1474)		*p*
	*n*	%	*n*	%		*n*	%	*n*	%	
Brain CT scan					0.205					<0.0001
No	2379	58.14	1857	56.67		1803	58.39	619	41.99	
Yes	1713	41.86	1420	43.33		1285	41.61	855	58.01	
Time from ED arrival to brain CT scan (minute), median (IQR)	30	(20–45)	22	(14–35)	<0.0001	27	(18–42)	21	(13–34)	<0.0001
Brain operation					0.459					0.020
No	4057	99.14	3254	99.30		3066	99.29	1453	98.58	
Yes	35	0.86	23	0.70		22	0.71	21	1.42	
Time from ED arrival to brain operation (hour), median (IQR)	6	(3–15)	4	(2–6)	0.174	5	(3–8)	5	(3–7)	0.788

IQR, interquartile range. CT, computed tomography; TBI, traumatic brain injury; COVID-19, coronavirus disease 2019; P1, January to 30 April 2019; P2, 11 May to 31 July 2021. Pre-pandemic period refers to the same span from 2019.

## Data Availability

The data that support the findings of this study are available from the corresponding author upon reasonable request.

## Supplementary Materials

The following are available online at https://www.mdpi.com/article/10.3390/jcm10225314/s1, Figure S1: The distribution of the intracranial injuries (S06.0–S06.9) between the COVID-19 pandemic era and the pre-COVID-19 pandemic era during (**A**) P1 and (**B**) P2, Table S1: The neurosurgical procedures and its frequency in the pre-pandemic and pandemic periods.
