# Supplementary material for: Effects of the COVID-19 Pandemic on Treatment Efficiency for Traumatic Brain Injury in the Emergency Department: A Multicenter Study in Taiwan
Source: J Clin Med. 2021 Nov 15;10(22):5314. doi: 10.3390/jcm10225314 (PMC8621260; doi:10.3390/jcm10225314)
Supplement: Supplementary file 1 [file jcm-10-05314-s001.zip › Table S1.pdf]

Table S1. The neurosurgical procedures and its frequency in the pre-pandemic and pandemic periods.

**Neurosurgical procedures (no.)**	**Pre-pandemic period corresponding to P1**	**P1**	**Pre-pandemic period corresponding to P2**	**P2**
Removal of epidural hematoma	1	1	0	0
Removal of subdural hematoma	8	0	5	3
ICP monitoring	0	1	4	2
External ventricular drainage	0	1	0	0
Removal of epidural hematoma and ICP monitoring	5	4	1	0
Removal of subdural hematoma and ICP monitoring	9	6	6	10
Removal of intracerebral hematoma and ICP monitoring	4	3	1	1
External ventricular drainage and ICP monitoring	1	0	0	1
Removal of epidural hematoma, subdural hematoma and ICP monitoring	0	2	0	0
Removal of epidural hematoma, intracerebral hematoma, and ICP monitoring	0	0	0	1
Removal of subdural hematoma, intracerebral hematoma, and ICP monitoring	1	3	1	1
Removal of epidural hematoma, craniectomy, and ICP monitoring	1	0	0	0
Removal of subdural hematoma, craniectomy, and ICP monitoring	1	0	1	0
Removal of intracerebral hematoma, craniectomy, and ICP monitoring	1	1	0	0

Removal of subdural hematoma, external ventricular drainage, and ICP monitoring	3	1	0	1
Removal of intracerebral hematoma, external ventricular drainage, and ICP monitoring	0	0	3	0
Removal of subdural hematoma, intracerebral hematoma, external ventricular drainage, and ICP monitoring	0	0	0	1

ICP, Intracranial Pressure. P1, January to April 30, 2019; P2, May 11 to July 31, 2021. Pre-pandemic period refers to the same span from 2019.
