# Supplementary figures and images for: Effects of the COVID-19 Pandemic on Treatment Efficiency for Traumatic Brain Injury in the Emergency Department: A Multicenter Study in Taiwan
Source: J Clin Med. 2021 Nov 15;10(22):5314. doi: 10.3390/jcm10225314 (PMC8621260; doi:10.3390/jcm10225314)

A

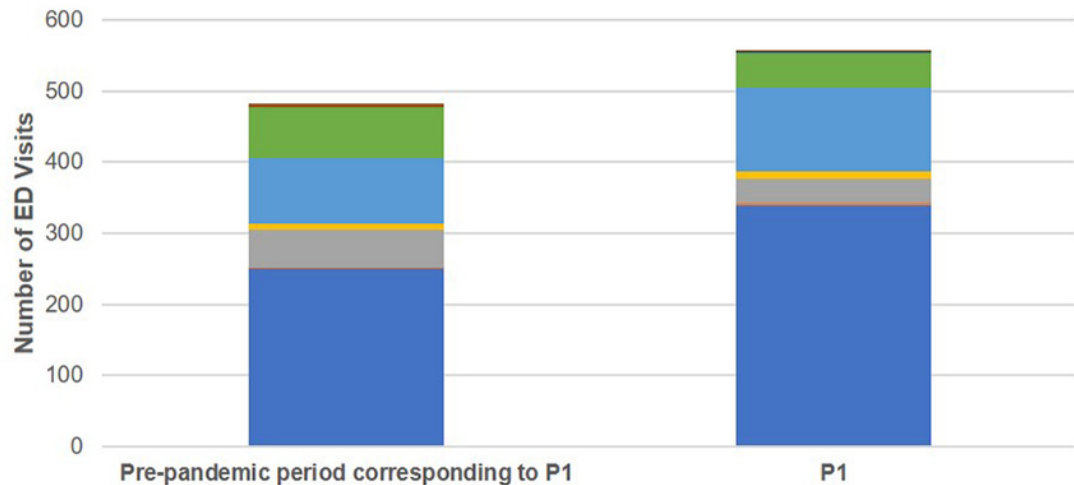

B

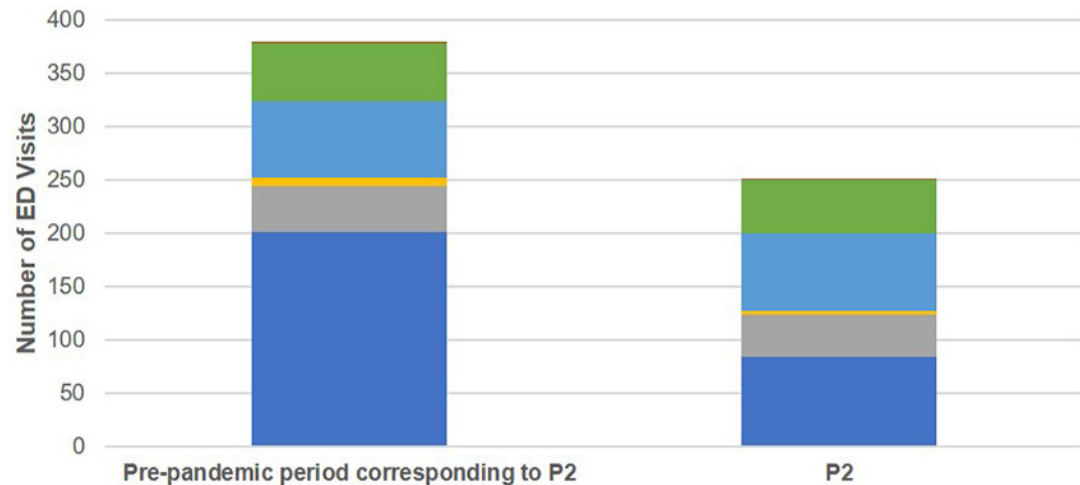

Supplement: Supplementary file 1 [file jcm-10-05314-s001.zip › Figure S1.pdf]
